# A Systematic Review of In-Home Smart Technology Adoption to Improve Older Adult Health and Family Caregiving

**DOI:** 10.1093/geroni/igaa057.1318

**Published:** 2020-12-16

**Authors:** Geunhye Park, Erin Robinson

**Affiliations:** University of Missouri-Columbia, Columbia, Missouri, United States

## Abstract

In-home and internet-based smart technologies to improve older adult health has been rapidly developing. Technologies such as in-home sensors and smart homes enable older adults to live independently and age in place. These technologies also assist informal caregivers in their roles, thus reducing caregiver burden. However, technology adoption among older adults and family caregivers has been relatively low and reasons for technology acceptance are complex. Therefore, the purpose of this study was to conduct a systematic review of the literature, examining acceptance and adoption of in-home, internet-based smart technologies that are designed to improve health outcomes of older adults and can assist family caregivers in providing supports. This study utilized the Rew method (2011) and included peer-reviewed research articles published between 1991 and 2019 and available in: ISI Web of Science; PubMed; Scopus; CINAHL; and PsycInfo. A total of 1,227 relevant articles were identified with the search strings used and a final sample of 48 articles were included after the title, abstract, and full article review processes. Findings highlight several facilitators and barriers to technology adoption. Some facilitators to adoption include: technology familiarity, safety/security, personally tailored, non-obtrusive design, easy access, and reduction of caregiver burden. A few barriers include: cost, difficulty to use, time, stigma, privacy, data accuracy, and confidence. Additional findings will also be presented. A more thorough understanding of these facilitators and barriers to acceptance/adoption is crucial for the successful dissemination of in-home, internet-based smart technologies. Increased adoption can improve older adult health and reduce caregiver burden.

